# High SPINK4 Expression Predicts Poor Outcomes among Rectal Cancer Patients Receiving CCRT

**DOI:** 10.3390/curroncol28040218

**Published:** 2021-06-25

**Authors:** Tzu-Ju Chen, Yu-Feng Tian, Chia-Lin Chou, Ti-Chun Chan, Hong-Lin He, Wan-Shan Li, Hsin-Hwa Tsai, Chien-Feng Li, Hong-Yue Lai

**Affiliations:** 1 Department of Pathology, Chi Mei Medical Center, Tainan 710, Taiwan; a108n2@mail.chimei.org.tw (T.-J.C.); a61101@mail.chimei.org.tw (H.-L.H.); a80818@mail.chimei.org.tw (W.-S.L.); livelychord@yahoo.com.tw (H.-H.T.); 2 Department of Medical Technology, Chung Hwa University of Medical Technology, Tainan 717, Taiwan; 3 Institute of Biomedical Sciences, National Sun Yat-Sen University, Kaohsiung 804, Taiwan; 4 Division of Colon and Rectal Surgery, Department of Surgery, Chi Mei Medical Center, Tainan 710, Taiwan; van0112@hotmail.com (Y.-F.T.); clchou3@gmail.com (C.-L.C.); 5 Department of Medical Research, Chi Mei Medical Center, Tainan 710, Taiwan; h67350@gmail.com; 6 National Institute of Cancer Research, National Health Research Institutes, Tainan 704, Taiwan; 7 Department of Optometry, Chung Hwa University of Medical Technology, Tainan 717, Taiwan; 8 Institute of Precision Medicine, National Sun Yat-Sen University, Kaohsiung 804, Taiwan; 9 Department of Pathology, School of Medicine, College of Medicine, Kaohsiung Medical University, Kaohsiung 807, Taiwan

**Keywords:** SPINK4, rectal cancer, chemoradiotherapy, metastasis, ECM

## Abstract

Background: Patients with rectal cancer can prospectively be favored for neoadjuvant concurrent chemoradiotherapy (CCRT) to downstage before a radical proctectomy, but the risk stratification and clinical outcomes remain disappointing. Methods: From a published rectal cancer transcriptome dataset (GSE35452), we highlighted extracellular matrix (ECM)-linked genes and identified the serine protease inhibitor Kazal-type 4 (SPINK4) gene as the most relevant among the top 10 differentially expressed genes associated with CCRT resistance. We accumulated the cases of 172 rectal cancer patients who received neoadjuvant CCRT followed by surgery and collected tumor specimens for the evaluation of the expression of SPINK4 using immunohistochemistry. Results: The results revealed that high SPINK4 immunoexpression was significantly related to advanced pre-CCRT and post-CCRT tumor status (both *p* < 0.001), post-CCRT lymph node metastasis (*p* = 0.001), more vascular and perineurial invasion (*p* = 0.015 and *p* = 0.023), and a lower degree of tumor regression (*p* = 0.001). In univariate analyses, high SPINK4 immunoexpression was remarkably correlated with worse disease-specific survival (DSS) (*p* < 0.0001), local recurrence-free survival (LRFS) (*p* = 0.0017), and metastasis-free survival (MeFS) (*p* < 0.0001). Furthermore, in multivariate analyses, high SPINK4 immunoexpression remained independently prognostic of inferior DSS and MeFS (*p* = 0.004 and *p* = 0.002). Conclusion: These results imply that high SPINK4 expression is associated with advanced clinicopathological features and a poor therapeutic response among rectal cancer patients undergoing CCRT, thus validating the prospective prognostic value of SPINK4 for those patients.

## 1. Introduction

Colorectal cancer (CRC) is the third most common cancer and the second major cause of cancer-associated deaths globally [1]. Adenocarcinoma of the rectum accounts for around one-third of all CRCs, and the incidence of rectal adenocarcinoma is 50% or more among Asian populations [2]. The extensive use of a multimodality therapy strategy involving preoperative neoadjuvant concurrent chemoradiotherapy (CCRT), and later total mesorectal excision, is the currently recommended regimen for patients who are initially staged with T3 or T4 rectal cancer or for whom perirectal lymph node metastasis is suspected. Long-term evaluation has shown that preoperative adjuvant therapy contributes to reduced local recurrence [3]; however, the reduction in mortality has slowed due to the high rate of distant metastasis [4] for rectal cancer. This situation draws attention to the necessity for the conception of better therapeutic strategies focused on controlling elusive micrometastases.

With the implementation of precision medicine, recent studies have supported the application of genetic biomarkers to enable better risk stratification and predict clinical outcomes. This offers clinicians the opportunity to individually tailor early interventions, which would help optimize therapy. Tumors leverage extracellular matrix (ECM) remodeling to create a microenvironment that promotes tumorigenesis and metastasis. In response to pathological triggers, ECM-degrading matrix metalloproteinases (MMPs) and serine and cysteine proteases are released to remodel the ECM, which is necessary for cancer cell metastasis and invasion. Unfortunately, most clinical trials using MMP inhibitors thus far have been disappointing [5]. One of the explanations for the failure of these clinical trials is the inappropriate use of broad-spectrum MMP inhibitors; some MMPs exert tumor-suppressing effects [6], thus indicating that more specific biomarkers need to be evaluated.

The serine protease inhibitor Kazal-type 4 (SPINK4), also called PEC60, was initially isolated from the intestine of a pig [7] and is expressed mainly in the gastrointestinal tract and immune system [8]. The SPINK4 gene—mapped to chromosome 9p13.3 in humans—encodes an 86-amino acid precursor protein consisting of a 26-amino acid signal sequence, which is characterized by C-terminal cysteine, N-terminal glutamic acid, and a total of 60 residues secreted from cells [8]. As a protease inhibitor, SPINK4 is believed to participate in the defense against the proteolytic degradation of mucosal and epithelial tissues. Interestingly, it has been suggested that the serum SPINK4 level is increased in colorectal cancer patients and has high diagnostic utility [9], whereas the expression of SPINK4 has been reported to be reduced in colorectal cancer tumor specimens and associated with poor survival [10]. However, the expression of SPINK4 in tumor tissues from patients receiving neoadjuvant CCRT, as well as its practical importance—especially for rectal cancer—are largely unrevealed.

## 2. Materials and Methods

### 2.1. An Evaluation of the Gene Expression Profiles in Rectal Cancer

To survey the prospective genes connected with the response to CCRT, a public transcriptome dataset (GSE35452) from the GEO database (NCBI, Bethesda, MD, USA), which incorporated 46 tumor specimens from rectal cancer patients who underwent neoadjuvant CCRT, was analyzed. To computerize expression levels, the raw CEL files of the Affymetrix Human Genome U133 Plus 2.0 microarray platform were imported into the Nexus Expression 3 statistical software (BioDiscovery, El Segundo, CA, USA) to analyze all probes without filtering. Two-tailed tests were performed, and *p* < 0.05 was considered to be the criterion for statistical significance. The tumor specimens were divided into groups of “responders” and “nonresponders”, as determined by the response to neoadjuvant CCRT. Under supervision, the statistical significance of each transcript was examined by comparing responders to nonresponders; those with a log2-transformed expression fold change >0.1 and a *p*-value < 0.01 were picked out for further analysis. Functional profiling of the top 10 differentially expressed genes associated with CCRT resistance was performed using the Gene Ontology (GO) database [11], based on biological processes and/or molecular functions. The enrichment was also analyzed based on GO terms by using the Nexus Expression 3 software (BioDiscovery) to identify expression alterations across the whole transcriptome.

### 2.2. Patient Enrollment

This study was approved by the Institutional Review Board of Chi Mei Medical Center (10302014). We gathered a total of 172 rectal cancer patients with formalin-fixed paraffin-embedded (FFPE) tissue specimens, as previously described [12]. The initial clinical stage was decided through imaging tests, and those who were initially diagnosed with distant metastasis were ruled out. All patients were administered a 24 h continuous infusion of 5-fluorouracil-based chemotherapy concomitant with radiation (45–50 Gy) in 25 fractions over a period of 5 weeks, accompanied by a curative proctectomy after 4 weeks. Adjuvant chemotherapy was given for those with either a nodal status beyond N1 or a pre-CCRT or post-CCRT tumor status beyond T3. All patients were routinely monitored after diagnosis until death or the last follow-up.

### 2.3. Histopathological and Immunohistochemical Assessments

The tumor specimens were reviewed by two expert pathologists (Wan-Shan Li and Chien-Feng Li) who were blinded to patient clinical information, and the T and N stages were decided in agreement with the 7th AJCC TNM staging system. The tumor regression grade was assessed in accordance with the interpretation provided by Dworak et al. [13]. The immunohistochemical staining was conducted as previously described [14] and probed with an anti-SPINK4 antibody (PA5-81038, 1:500) (Thermo Fisher Scientific, Waltham, MA, USA). The SPINK4 immunoreactivity was assessed using the H-score, which is calculated through the following equation: H-score = Σ*Pi* (*i* + 1), where *i* is the intensity of stained tumor cells (0 to 3+) and *Pi* is the percentage of staining for each intensity (ranging from 0% to 100%). If there were scoring variances, the two pathologists reviewed the slides at the same time and generated a consistent H-score. We defined tumors with H-scores above or equal to the median of all scored cases as having high SPINK4 expression.

### 2.4. Statistical Analysis

The associations of SPINK4 expression with clinicopathological characteristics were assessed through χ^2^ tests. The Kaplan–Meier method was used for survival analysis, and the log-rank test was used to calculate the interval from the operation to the event of interest. The univariate analyses of the variables that revealed prognostic significance were incorporated into the Cox multivariate regression analysis adjusting for potential confounding parameters to identify independent prognostic factors. The statistical analyses were conducted using SPSS software version 22.0 (IBM Corporation, Armonk, NY, USA), with a *p*-value of less than 0.05 considered to be statistically significant.

## 3. Results

### 3.1. SPINK4 Gene Upregulation Is Predictive of Poor Response to CCRT in Rectal Adenocarcinoma

**Figure 1:**
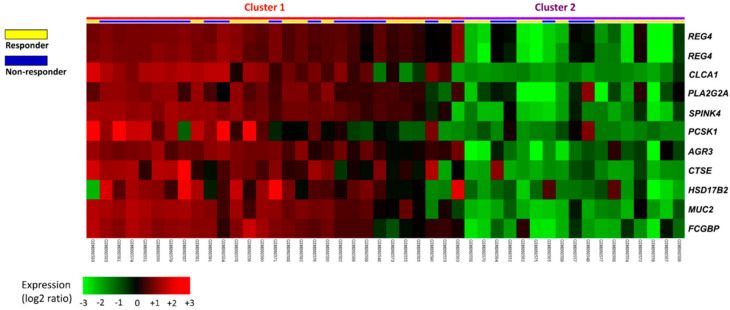
Expression profiles of the top 10 differentially expressed genes linked to CCRT resistance from a public transcriptome dataset (GSE35452) in the GEO database. All probes were analyzed without preselection. The statistical significance of each transcript was examined by comparing responders to nonresponders. The expression levels of upregulated and downregulated genes are marked in red and green, respectively. The SPINK4 gene was identified as one of the most significantly upregulated genes among CCRT nonresponders.

To survey the prospective biomarkers of rectal cancer cells responsive to preoperative CCRT, a published rectal cancer transcriptome dataset (GSE35452) was applied for data mining, incorporating 46 patients undergoing neoadjuvant CCRT followed by standardized curative resection. Twenty-four patients (52.2%) showing a response to CCRT were categorized as responders, whereas 22 patients (47.8%) showing resistance to CCRT were classified as nonresponders. Eleven probes, covering the top 10 transcripts associated with CCRT resistance in rectal carcinoma, were identified (Table 1 and Figure 1). We chose SPINK4 for further investigation, as it is located in the extracellular space and plays a role in ECM remodeling. The results revealed that SPINK4 gene expression was considerably upregulated among CCRT nonresponders (*p* = 0.0001), thus prompting further analysis to elucidate the role of SPINK4 in rectal cancer.

### 3.2. Clinicopathological Features of Our Rectal Cancer Cohort

We enrolled 172 rectal cancer patients, comprising 64 women (37.2%) and 108 men (62.8%), with a median age of 63, varying from 22 to 88 (Table 2). During pre-CCRT clinical staging, the depth of invasion was restricted to the muscularis propria (cT1-2) in 81 cases (47.1%), and the nodal status was cN0 in 125 cases (72.7%). The invasive depth was pathologically restricted to the muscularis propria (ypT1-2) in 86 cases (50%), and there was no locoregional lymph node metastasis (ypN0) in 123 cases (71.5%) following CCRT treatment. Perineurial invasion and vascular invasion were detected in 5 (2.9%) and 15 (8.7%) tumors, respectively. The tumor regression grade was used for the evaluation of tumor response to CCRT and varied from 0 to 4, including grade 0–1 (*n* = 37, 21.5%), grade 2–3 (*n* = 118, 68.6%), and grade 4 (*n* = 17, 9.9%).

### 3.3. Associations between SPINK4 Expression and Clinicopathological Characteristics

**Figure 2:**
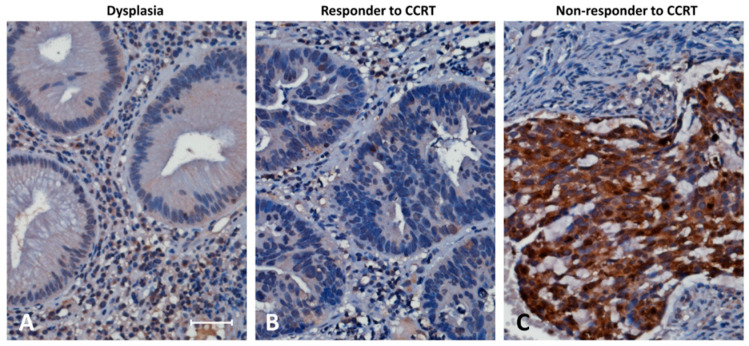
Higher SPINK4 immunoexpression was observed among CCRT nonresponders. Immunohistochemical staining was performed with an anti-SPINK4 antibody. Rectal dysplasia (**A**) revealed no expression of SPINK4. Tumor tissues showed low SPINK4 immunoexpression among CCRT responders and (**B**) high SPINK4 immunoexpression among CCRT nonresponders. (**C**) Scale bar, 100 µm.

The immunohistochemical staining showed that SPINK4 immunoreactivity was significantly higher among CCRT nonresponders (Figure 2). Table 2 reveals the correlations of SPINK4 immunoexpression with the clinicopathological variables. Low SPINK4 expression was remarkably linked with early pre-CCRT and post-CCRT tumor status (both *p* < 0.001), post-CCRT negative nodal status (*p* = 0.001), and less perineurial and vascular invasion (*p* = 0.023 and *p* = 0.015). Additionally, following CCRT treatment, low SPINK4 expression was remarkably linked with a greater level of tumor regression (*p* = 0.001).

### 3.4. Survival Analysis and Prognostic Utility of SPINK4 Expression

**Figure 3:**
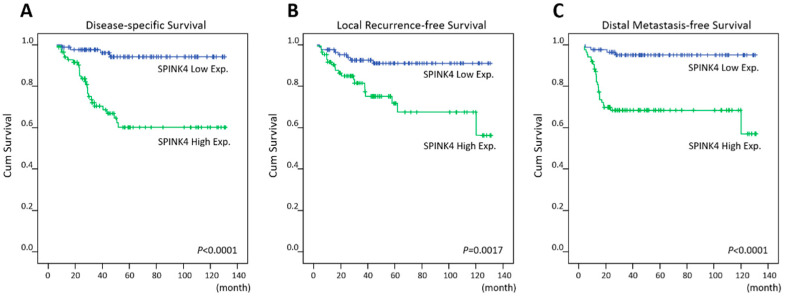
Survival analysis. Kaplan–Meier plots showed that high SPINK4 immunoexpression was significantly linked to worse (**A**) disease-specific survival, (**B**) local recurrence-free survival, and (**C**) metastasis-free survival.

Low SPINK4 expression was significantly correlated with better disease-specific survival (DSS) (*p* < 0.0001), local recurrence-free survival (LRFS) (*p* = 0.0017), and metastasis-free survival (MeFS) (*p* < 0.0001) in univariate analysis (Table 3 and Figure 3). High tumor regression grades and early post-CCRT tumor status were remarkably linked with longer DSS, LRFS, and MeFS (all *p* < 0.009). Pre-CCRT lymph node metastasis was considerably linked only with shorter LRFS (*p* = 0.007). Vascular invasion was prognostic of worse DSS and LRFS (*p* = 0.0184 and *p* = 0.0028). Low SPINK4 expression remained significantly prognostic of better DSS and MeFS (*p* = 0.004 and *p* = 0.002) following multivariate analysis (Table 4). Lower tumor regression grade was considerably linked with inferior LRFS and MeFS (*p* = 0.018 and *p* = 0.028). Vascular invasion was remarkably correlated only with worse LRFS (*p* = 0.028).

## 4. Discussions

There are four original members of the SPINK family in humans (SPINK1, SPINK2, SPINK4, and SPINK5). SPINK1 is expressed largely in the pancreas, gastrointestinal tract, and urinary system and has been suggested to promote pancreatic cancer [15], colorectal cancer [16], and prostate cancer [17] progression. Moreover, SPINK1 is involved in tumor metastasis, acts as a prognostic biomarker for lung cancer [18], and acts as a predictive biomarker for ovarian cancer [19]. Additionally, SPINK1 is reported to repress granzyme A- and serine protease-induced cell apoptosis and confer resistance to chemotherapy [20,21]. SPINK2 is detected in the seminal vesicle and testis, where its antimicrobic function may be associated with infertility [22]. SPINK5 is expressed in the skin and tonsil and has been linked with atopic dermatitis and asthma [23]. SPINK4, as a gastrointestinal peptide, was originally recognized for its suppression of glucose-induced insulin secretion [8]; however, few previous reports have focused on the role of SPINK4 in tumors. Here, we provide the first evidence demonstrating that high SPINK4 expression is significantly related to poor clinical outcomes, and functions as a prognostic biomarker for rectal cancer patients receiving neoadjuvant CCRT.

Dysfunctional glucose-mediated insulin release is a characteristic of type 2 diabetes, and induces hyperglycemia [24]. Hyperglycemia causes insulin resistance [25], which then triggers a compensatory mechanism that increases insulin levels, thus leading to hyperinsulinemia. Insulin supports tumorigenesis and reduces the transport of chemotherapeutic agents to the tumor by changing the microvasculature [26]. Moreover, several studies have demonstrated that diabetes is a risk factor for rectal cancer and is linked to poor outcomes [27], and others have established that the efficacy of neoadjuvant CCRT is poor in diabetic patients [28]. Previous studies have also shown that hyperinsulinemia is related to a higher risk for Alzheimer’s disease [29], and vice versa [30], and that high expression of SPINK4 is significantly related to biological processes in Alzheimer’s disease [10]. However, the correlations among SPINK4 expression, diabetes, and chemoresistance in rectal cancer require further identification. Interestingly, Wang et al. reported that SPINK4 expression was downregulated in CRC tumor specimens and was associated with poor survival [10]; however, none of the enrolled patients underwent preoperative radiation or chemotherapy in this study. The current chemotherapy regimen for preoperative rectal cancer patients is the use of 5-fluorouracil (5-FU)-based agents. 5-FU is a pyrimidine analog that can be misincorporated into nucleic acids in place of uracil or thymine to restrict enhanced pyrimidine synthesis and decrease proliferation in cancer cells. Urea cycle dysregulation-altering nitrogen utilization for pyrimidine synthesis is associated with a transversion bias known to generate immunogenic neoantigens [31], suggesting a worse prognosis but a better response to immunotherapy. Analogously, low SPINK4 expression might be associated with poor prognosis in CRC patients, but might also be associated with improved outcomes among rectal cancer patients undergoing neoadjuvant CCRT. However, whether SPINK4 expression correlates with 5-FU, and even immunotherapy efficacy and the underlying mechanisms involved in this process, are still obscure and warrant further dissection. In addition, CRC is a heterogeneous disease, and the structure and mutational signature between the colon and rectum are quite different [32]. Therefore, the different, and sometimes paradoxical, expression of SPINK4 appears to be dependent on cell type-specific contexts, providing another explanation for why our results from this rectal cancer cohort seem distinct from those of Wang’s study [10] in a mixed colon and rectal cancer cohort.

Using the STRING database [33], we identified HtrA serine peptidase 1 (HTRA1) as one of the top SPINK4-interacting proteins. HTRA1 has been suggested to participate in chemotherapy-induced cytotoxicity and has been proposed to be a tumor suppressor [34]. Interestingly, high HTRA1 expression is associated with shorter survival in renal and urothelial cancers, as evaluated by the Human Protein Atlas database [35]; conversely, HTRA1 tends to be downregulated in the metastatic foci of melanoma and gastric cancer when compared to the primary tumor [36,37]. The aforementioned results imply an inverse relationship between SPINK4 and HTRA1 expression. In addition, the downregulation of HTRA1 also correlates with chemoresistance in colon cancer through the activation of the PI3K/AKT pathway [38]. Therefore, whether SPINK4 can induce chemoresistance and metastasis indirectly through serine protease HTRA1 inhibition in rectal cancer deserves further verification.

To further predict the biological functions of SPINK4, the top 200 genes co-upregulated *(*Table S1*)* or co-downregulated *(*Table S2*)* with SPINK4 in colorectal adenocarcinoma from the TCGA database (*n* = 594) were evaluated. Using the PANTHER annotation system, we identified the maintenance of the gastrointestinal epithelium (GO: 0030277, fold enrichment: 25.49) and epithelial structure maintenance (GO: 0010669, fold enrichment: 18.88) as 2 of the top 10 GO terms associated with SPINK4 upregulation (Figure S1); we further identified the mucin 2 (MUC2) gene involved in these 2 biological processes, thus suggesting that SPINK4 can maintain gastrointestinal epithelium structure. Impressively, the MUC2 gene is the third most positively correlated with SPINK4 (Spearman’s correlation: 0.8) *(*Figure S2*)* and one of the top 10 genes associated with CCRT resistance ***(***Table 1*)*. However, whether SPINK4 can orchestrate MUC2 to form a defensive barrier against chemoradiotherapy, as well as the exact molecular mechanisms involved in this process, require further analysis.

Chemotherapy and radiation not only trigger cancer cell apoptosis but also induce an environment conducive to tumor recurrence and metastasis. In CRC, metastasis-specific mutations are enhanced in phosphatidylinositol 3-kinase (PI3K)/AKT signaling, cell adhesion, and ECM, implying genetic programming for specific recombination and colonization [39]. SPINK1 and SPINK3 have been suggested to promote the proliferation of colorectal cancer cells [16] and rat liver cells [40], respectively, through the PI3K/AKT signaling pathway. As mentioned previously, whether SPINK4 can downregulate HTRA1 to activate the PI3K/AKT pathway involved in rectal cancer metastasis needs further verification. L1 cell adhesion molecule (L1CAM) confers metastasis-initiating abilities and chemoresistance in CRC [41] and radioresistance in ovarian cancer [42]. It has been reported that brain metastatic cells from breast cancer and lung cancer induce large amounts of serine protease inhibitors (SERPINs) to prevent plasminogen activator (PA) destruction of L1CAM and mediate the spread of metastatic cells [43]. SPINK4 has also been demonstrated to play a role in the brain [44]. Nevertheless, more studies are required to verify whether SPINK4 can activate L1CAM through PA inhibition to drive metastasis and CCRT resistance in rectal cancer. Plasminogen activator inhibitor-1 (PAI-1), also called SERPINE1, functions as an inhibitor of urokinase-type plasminogen activator (uPA) and was originally associated with thrombosis. It has been demonstrated that both tumor invasion and angiogenesis are impaired in PAI-1-deficient transgenic mice [45]. Because uPA binding to the uPA receptor increases the binding affinity of vitronectin to the uPA receptor [46], a blockade of PAI-1 can reduce cancer cell migration, proliferation, and survival by increasing cell adhesion to the ECM through the promotion of uPA receptor binding to vitronectin [47,48]. Since numerous SPINK members also act as inhibitors of serine protease uPA [49,50,51], whether SPINK4 can dissociate the binding between matrix-bound vitronectin and the uPA receptor to create a metastatic niche in rectal cancer needs to be confirmed.

The current research still has several restrictions. First, rectal cancer patients treated with preoperative CCRT were analyzed retrospectively at a single institution. Second, the exertion of SPINK4 function in cancer progression is generally indirect and not straightforward. Accordingly, further analysis is required to dissect the detailed molecular mechanisms underlying the chemoradioresistant and metastatic effects of SPINK4 in rectal cancer. Third, it is not useful for clinical assessment if we do not define the optimal threshold for high SPINK4 expression. Finally, the number of samples was not sufficient; consequently, the value of SPINK4 expression should be verified by prospective multicenter studies.

## 5. Conclusions

Our present investigation has revealed that high SPINK4 expression is connected to aggressive rectal cancer features and is a unique prognostic biomarker of inferior patient outcomes for those undergoing neoadjuvant CCRT.

## Figures and Tables

**Table 1:** Summary of the top 10 differentially expressed genes associated with CCRT resistance in rectal carcinoma.

Probe	ComparisonLog Ratio	Comparison *p*-Value	Gene Symbol	Gene Name	Biological Process	Molecular Function
223447_at	2.9382	<0.0001	*REG4*	regenerating islet-derived family; member 4		sugar-binding
1554436_a_at	2.9364	<0.0001				
210107_at	2.1851	0.0001	*CLCA1*	chloride channel; calcium-activated; family member 1	integral to plasma membrane	chloride channel activity
203649_s_at	1.9828	<0.0001	*PLA2G2A*	phospholipase A2; group IIA (platelets; synovial fluid)	endoplasmic reticulum, extracellular region, membrane	calcium ion-binding, calcium-dependent phospholipase A2 activity, hydrolase activity, metal ion-binding, phospholipase A2 activity, protein-binding
207214_at	1.891	0.0001	*SPINK4*	serine peptidase inhibitor; Kazal-type 4		endopeptidase inhibitor activity, serine-type endopeptidase inhibitor activity
205825_at	1.8447	<0.0001	*PCSK1*	proprotein convertase subtilisin/kexin-type 1	cytoplasmic vesicle	calcium ion-binding, hydrolase activity, peptidase activity, proprotein convertase 1 activity, serine-type endopeptidase activity, subtilase activity
228241_at	1.8125	<0.0001	*AGR3*	anterior gradient homolog 3 (Xenopus laevis)		
205927_s_at	1.7848	<0.0001	*CTSE*	cathepsin E	endosome	aspartic-type endopeptidase activity, cathepsin E activity, hydrolase activity, pepsin A activity, peptidase activity
204818_at	1.6874	<0.0001	*HSD17B2*	hydroxysteroid (17-beta) dehydrogenase 2	endoplasmic reticulum membrane, integral to membrane, membrane	estradiol 17-beta-dehydrogenase activity, oxidoreductase activity
204673_at	1.6574	0.0002	*MUC2*	mucin 2; oligomeric mucus/gel-forming	extracellular region, extracellular space, proteinaceous extracellular matrix	extracellular matrix constituent; lubricant activity, extracellular matrix structural constituent
203240_at	1.5838	0.0004	*FCGBP*	Fc fragment of IgG binding protein	membrane	chloride channel activity

**Table 2:** Correlations between SPINK4 expression and clinicopathological features in 172 rectal cancer patients receiving neoadjuvant CCRT.

Parameter		No.	SPINK4 Expression		*p*-Value
			Low Exp.	High Exp.	
Gender	Male	108	48	60	0.058
	Female	64	38	26	
Age	<70	106	49	57	0.210
	≧70	66	37	29	
Pre-Tx tumor status (Pre-T)	T1–T2	81	52	29	<0.001 *
	T3–T4	91	34	57	
Pre-Tx nodal status (Pre-N)	N0	125	66	59	0.231
	N1–N2	47	20	27	
Post-Tx tumor status (Post-T)	T1–T2	86	62	24	<0.001 *
	T3–T4	86	24	62	
Post-Tx nodal status (Post-N)	N0	123	71	52	0.001 *
	N1–N2	49	15	34	
Vascular invasion	Absent	157	83	74	0.015 *
	Present	15	3	12	
Perineurial invasion	Absent	167	86	81	0.023 *
	Present	5	0	5	
Tumor regression grade	Grade 0–1	37	10	27	0.001 *
	Grade 2~3	118	63	55	
	Grade 4	17	13	4	

Tx, treatment; * statistically significant.

**Table 3:** Univariate log-rank analyses for important clinicopathological variables and SPINK4 expression.

Parameter		No. of Cases	DSS		LRFS		MeFS	
			No. of Events	*p*-Value	No. of Events	*p*-Value	No. of Events	*p*-Value
Gender	Male	108	20	0.9026	7	0.2250	17	0.3520
	Female	64	11		20		14	
Age	<70	106	19	0.8540	18	0.6615	20	0.7427
	≧70	66	12		9		11	
Pre-Tx tumor status (Pre-T)	T1–T2	81	10	0.0776	10	0.2261	11	0.1745
	T3–T4	91	21		17		20	
Pre-Tx nodal status (Pre-N)	N0	125	19	0.0711	15	0.0070 *	19	0.0973
	N1–N2	47	21		12		12	
Post-Tx tumor status (Post-T)	T1–T2	86	7	0.0006 *	7	0.0040 *	8	0.0033 *
	T3–T4	86	24		20		23	
Post-Tx nodal status (Post-N)	N0	123	21	0.5998	16	0.1320	20	0.4634
	N1–N2	49	10		11		11	
Vascular invasion	Absent	157	25	0.0184 *	21	0.0028 *	27	0.4470
	Present	15	6		6		4	
Perineurial invasion	Absent	167	29	0.2559	25	0.0940	30	0.9083
	Present	5	2		2		1	
Tumor regression grade	Grade 0–1	37	13	0.0038 *	10	0.0090 *	14	0.0006 *
	Grade 2~3	118	17		17		16	
	Grade 4	17	1		0		1	
Down stage after CCRT	Non-Sig.	150	29	0.1651	24	0.5961	30	0.0853
	Sig. (>=2)	22	2		3		1	
SPINK4 expression	Low Exp.	86	4	<0.0001 *	7	0.0017 *	4	<0.0001 *
	High Exp.	86	27		20		27	

DSS, disease-specific survival; LRFS, local recurrence-free survival; MeFS, metastasis-free survival; * statistically significant.

**Table 4:** Multivariate analyses.

Parameter	DSS			LRFS			MeFS		
	H.R	95% CI	*p*-Value	H.R	95% CI	*p*-Value	H.R	95% CI	*p*-Value
Tumor regression grade	1.869	0.951–3.717	0.069	2.506	1.174**–**5.376	0.018 *	2.155	1.085**–**4.292	0.028 *
SPINK4 expression	5.310	1.697–16.613	0.004 *	1.997	0.739–5.399	0.173	6.000	1.969–18.279	0.002 *
Vascular invasion	1.851	0.737–4.650	0.190	3.096	1.133–8.458	0.028 *	-	-	-
Post-Tx tumor status (Post-T)	1.517	0.610–3.772	0.370	1.639	0.616–4.356	0.322	1.233	0.515–2.952	0.638
Pre-Tx nodal status (Pre-N)	-	-	-	0.844	0.364–1.958	0.693	-	-	-

DSS, disease-specific survival; LRFS, local recurrence-free survival; MeFS, metastasis-free survival; * statistically significant.

## Data Availability

Publicly available dataset was analyzed in this study. This data can be found here: https://www.ncbi.nlm.nih.gov/geo/query/acc.cgi?acc=GSE35452.

## Supplementary Materials

The following are available online at https://www.mdpi.com/article/10.3390/curroncol28040218/s1, Figure S1: The biological processes enriched in SPINK4 upregulation and downregulation, Figure S2: Correlations between SPINK4 and MUC2 gene expression, Table S1: The top 200 genes positively correlated with SPINK4, Table S2: The top 200 genes negatively correlated with SPINK4.

## Availability of Data and Materials:

The dataset analyzed in the current study is available in a public transcriptome dataset (GSE35452) from the GEO database (National Center for Biotechnology Information, Bethesda, MD, USA).
