# Supplementary material for: High SPINK4 Expression Predicts Poor Outcomes among Rectal Cancer Patients Receiving CCRT
Source: Curr Oncol. 2021 Jun 25;28(4):2373–84. doi: 10.3390/curroncol28040218 (PMC8293060; doi:10.3390/curroncol28040218)
Supplement: Supplementary file 1 [file curroncol-28-00218-s001.zip › curroncol-1249149-supplementary.pdf]

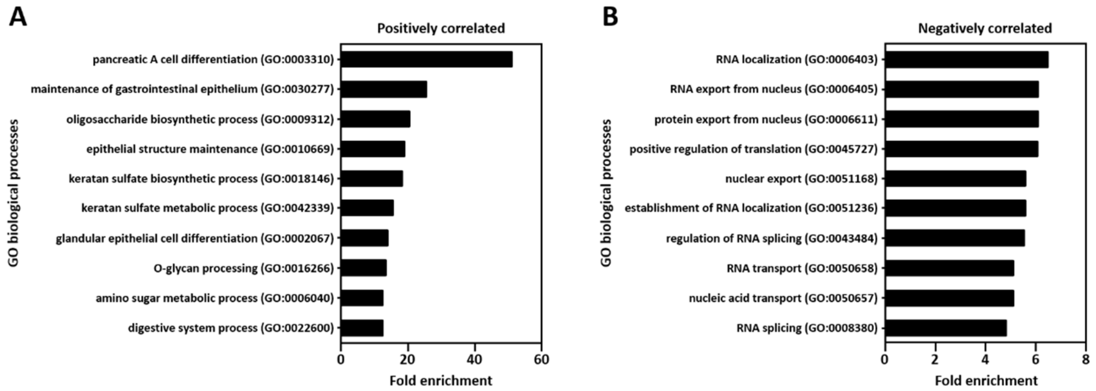

Figure S1. The biological processes enriched in *SPINK4* upregulation and downregulation. The genes co-expressed with *SPINK4* in colorectal adenocarcinoma in the TCGA database (n = 594) were surveyed using the cBioPortal online platform (http://cbioportal.org). The gene list (top 200 transcripts) from either (**A**) positive correlation or (**B**) negative correlation was further analyzed using PANTHER (http://pantherdb.org) according to biological process and ranked by fold enrichment for functional annotation.

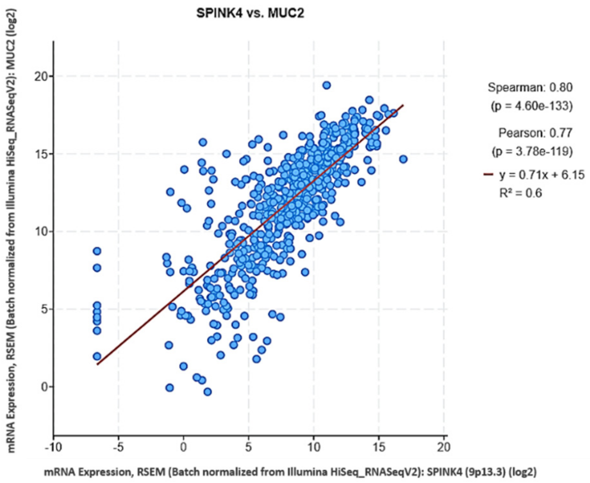

Figure S2. Correlations between *SPINK4* and *MUC2* gene expression. The data were exported from the TCGA database (n = 594) using the cBioPortal online platform.

Table S1. The top 200 genes positively correlated with *SPINK4*.

Correlated Gene	Cytoband	Spearman's Correlation	*p*-Value	*q*-Value
**B3GNT6**	11q13.5	0.86	1.28E-174	**2.57E-170**
**REG4**	1p12	0.814	1.79E-141	**1.79E-137**
**MUC2**	11p15.5	0.8	4.60E-133	**3.07E-129**
**HEPACAM2**	7q21.2	0.788	3.94E-126	**1.97E-122**
**FCGBP**	19q13.2	0.785	8.92E-125	**3.57E-121**
**REP15**	12p11.22	0.784	4.77E-124	**1.59E-120**
**ITLN1**	1q23.3	0.78	4.38E-122	**1.25E-118**
**FAM177B**	1q41	0.749	1.52E-107	**3.80E-104**
**SPDEF**	6p21.31	0.728	6.02E-99	**1.34E-95**
**ATOH1**	4q22.2	0.712	1.43E-92	**2.86E-89**
**FER1L6**	8q24.13	0.703	2.06E-89	**3.74E-86**
**CLCA1**	1p22.3	0.7	2.21E-88	**3.68E-85**
**ANO7**	2q37.3	0.692	1.47E-85	**2.26E-82**
**LRRC26**	9q34.3	0.681	4.90E-82	**7.00E-79**

**ST6GALNAC1**	17q25.1	0.674	1.64E-79	**2.19E-76**
**NEURL1**	10q24.33	0.662	5.53E-76	**6.92E-73**
**CAPN9**	1q42.2	0.661	1.42E-75	**1.67E-72**
**LINC00261**	20p11.21	0.649	3.84E-72	**4.27E-69**
**BEST2**	19p13.13	0.645	4.93E-71	**5.19E-68**
**TPSG1**	16p13.3	0.644	1.43E-70	**1.43E-67**
**GALNT8**	12p13.32	0.642	5.90E-70	**5.62E-67**
**CHST5**	16q23.1	0.64	1.26E-69	**1.14E-66**
**SLC18A1**	8p21.3	0.626	7.64E-66	**6.65E-63**
**CBFA2T3**	16q24.3	0.609	2.16E-61	**1.80E-58**
**ZG16**	16p11.2	0.608	3.86E-61	**3.09E-58**
**TMEM61**	1p32.3	0.608	4.28E-61	**3.30E-58**
**TOX**	8q12.1	0.607	8.26E-61	**6.13E-58**
**RAB26**	16p13.3	0.606	1.21E-60	**8.63E-58**
**ENTPD8**	9q34.3	0.602	1.11E-59	**7.66E-57**
**FFAR4**	10q23.33	0.601	2.52E-59	**1.68E-56**
**SLC4A4**	4q13.3	0.597	2.38E-58	**1.54E-55**
**AQP3**	9p13.3	0.594	1.18E-57	**7.39E-55**
**VSIG2**	11q24.2	0.593	2.18E-57	**1.33E-54**
**PTGER2**	14q22.1	0.585	1.31E-55	**7.69E-53**
**GALNTL6**	4q34.1	0.58	1.75E-54	**1.00E-51**
**ZBTB7C**	18q21.1	0.579	2.58E-54	**1.44E-51**
**BCAS1**	20q13.2	0.579	3.00E-54	**1.62E-51**
**KLF4**	9q31.2	0.578	5.66E-54	**2.98E-51**
**B4GALNT2**	17q21.32	0.577	7.76E-54	**3.98E-51**
**ATP2A3**	17p13.2	0.575	2.50E-53	**1.25E-50**
**IGFALS**	16p13.3	0.572	8.15E-53	**3.98E-50**
**CTSE**	1q32.1	0.571	1.48E-52	**7.07E-50**
**KLK3**	19q13.33	0.57	2.38E-52	**1.11E-49**
**SCGB2A1**	11q12.3	0.565	2.65E-51	**1.21E-48**
**AGR2**	7p21.1	0.565	3.35E-51	**1.49E-48**
**GFI1**	1p22.1	0.562	1.28E-50	**5.56E-48**
**RAP1GAP**	1p36.12	0.561	1.86E-50	**7.91E-48**
**KLK1**	19q13.33	0.559	7.12E-50	**2.97E-47**
**SERPINA1**	14q32.13	0.556	2.02E-49	**8.26E-47**
**KCNA6**	12p13.32	0.552	1.31E-48	**5.23E-46**
**MB**	22q12.3	0.547	1.42E-47	**5.56E-45**
**RASD1**	17p11.2	0.541	3.14E-46	**1.21E-43**
**KIF19**	17q25.1	0.54	5.10E-46	**1.93E-43**
**B3GNT7**	2q37.1|2q37.1	0.538	8.66E-46	**3.21E-43**
**SLITRK6**	13q31.1	0.535	4.61E-45	**1.68E-42**
**ITLN2**	1q23.3	0.534	5.12E-45	**1.83E-42**
**FABP2**	4q26	0.532	1.57E-44	**5.52E-42**
**LINC01550**	14q32.2	0.532	1.62E-44	**5.60E-42**
**NBPF7**	1p12	0.531	1.86E-44	**6.32E-42**
**TFF1**	21q22.3	0.53	4.01E-44	**1.34E-41**
**KLK12**	19q13.41	0.529	6.68E-44	**2.19E-41**
**CA2**	8q21.2	0.524	4.20E-43	**1.36E-40**
**NRAP**	10q25.3	0.523	8.58E-43	**2.73E-40**
**AGR3**	7p21.1	0.521	1.41E-42	**4.41E-40**
**CREB3L1**	11p11.2	0.518	5.61E-42	**1.73E-39**

**ASRGL1**	11q12.3	0.516	1.18E-41	**3.59E-39**
**ST3GAL4**	11q24.2	0.513	4.18E-41	**1.25E-38**
**KIAA1324**	1p13.3	0.51	1.89E-40	**5.56E-38**
**ANG**	14q11.2	0.502	4.34E-39	**1.26E-36**
**NKX2-2**	20p11.22	0.502	4.40E-39	**1.26E-36**
**XBP1**	22q12.1|22q12	0.501	6.01E-39	**1.69E-36**
**B3GALT5**	21q22.2	0.499	1.14E-38	**3.18E-36**
**MLPH**	2q37.3	0.497	2.44E-38	**6.68E-36**
**CANT1**	17q25.3	0.497	2.84E-38	**7.68E-36**
**RETNLB**	3q13.13	0.494	1.04E-37	**2.79E-35**
**C2ORF88**	2q32.2	0.492	1.83E-37	**4.82E-35**
**ST6GALNAC6**	9q34.11	0.49	4.12E-37	**1.07E-34**
**REG1A**	2p12	0.49	4.72E-37	**1.21E-34**
**FFAR2**	19q13.12	0.488	1.10E-36	**2.78E-34**
**VSIG1**	Xq22.3	0.486	2.15E-36	**5.37E-34**
**ALDH1L1**	3q21.3	0.485	3.13E-36	**7.75E-34**
**RHBDL3**	17q11.2	0.484	3.64E-36	**8.88E-34**
**MMP28**	17q12	0.484	4.49E-36	**1.08E-33**
**PTGDR2**	11q12.2	0.483	7.45E-36	**1.78E-33**
**PLAC8**	4q21.22	0.482	8.93E-36	**2.10E-33**
**MUC4**	3q29	0.481	1.19E-35	**2.77E-33**
**SMIM14**	4p14	0.481	1.21E-35	**2.78E-33**
**IL1R2**	2q11.2	0.479	2.51E-35	**5.72E-33**
**IMPA2**	18p11.21	0.478	3.77E-35	**8.49E-33**
**PLA2G2A**	1p36.13	0.478	3.87E-35	**8.62E-33**
**TRIM40**	6p22.1	0.476	9.02E-35	**1.98E-32**
**TRPA1**	8q21.11	0.475	1.33E-34	**2.90E-32**
**TSPAN1**	1p34.1	0.474	1.55E-34	**3.34E-32**
**CHGA**	14q32.12	0.474	2.01E-34	**4.28E-32**
**ERN2**	16p12.2	0.473	2.93E-34	**6.18E-32**
**HYAL1**	3p21.31	0.47	8.34E-34	**1.74E-31**
**FOXA3**	19q13.32	0.469	1.05E-33	**2.16E-31**
**GP2**	16p12.3	0.468	1.27E-33	**2.59E-31**
**MADCAM1**	19p13.3	0.468	1.33E-33	**2.69E-31**
**SGSM3**	22q13.1	0.467	1.99E-33	**3.98E-31**
**TFF3**	21q22.3	0.466	2.69E-33	**5.33E-31**
**CA8**	8q12.1	0.465	5.01E-33	**9.84E-31**
**C4BPB**	1q32.1	0.464	5.24E-33	**1.02E-30**
**DHRS9**	2q31.1	0.462	1.29E-32	**2.49E-30**
**L1TD1**	1p31.3	0.46	2.16E-32	**4.11E-30**
**TCN1**	11q12.1	0.458	5.31E-32	**1.00E-29**
**PLA2G10**	16p13.12	0.457	6.61E-32	**1.24E-29**
**RAB27A**	15q21.3	0.457	7.82E-32	**1.44E-29**
**RASD2**	22q12.3	0.457	7.82E-32	**1.44E-29**
**CLCA4**	1p22.3	0.456	8.55E-32	**1.56E-29**
**SLC28A2**	15q21.1	0.452	3.37E-31	**6.07E-29**
**AKAP5**	14q23.3	0.451	4.55E-31	**8.14E-29**
**KLK15**	19q13.33	0.45	7.54E-31	**1.34E-28**
**TC2N**	14q32.12	0.449	1.03E-30	**1.80E-28**
**FAM189A2**	9q21.12	0.447	2.13E-30	**3.70E-28**
**NXPE2**	11q23.2-q23.3	0.446	3.21E-30	**5.53E-28**

**GCG**	2q24.2	0.547	7.34E-30	**1.26E-27**
**RNASE4**	14q11.2	0.441	1.47E-29	**2.49E-27**
**RAB27B**	18q21.2	0.441	1.60E-29	**2.70E-27**
**PYY**	17q21.31	0.44	1.87E-29	**3.12E-27**
**TENT5A**	6q14.1	0.439	2.74E-29	**4.54E-27**
**ABHD3**	18q11.2	0.439	2.96E-29	**4.85E-27**
**ADTRP**	6p24.1	0.438	3.92E-29	**6.38E-27**
**SI**	3q26.1	0.538	9.32E-29	**1.50E-26**
**WFDC2**	20q13.12	0.435	9.71E-29	**1.56E-26**
**CATSPERB**	14q32.12	0.434	1.49E-28	**2.37E-26**
**KLK11**	19q13.41	0.434	1.52E-28	**2.40E-26**
**GSKIP**	14q32.2	0.433	1.75E-28	**2.74E-26**
**SDR16C5**	8q12.1	0.432	2.38E-28	**3.69E-26**
**AKR1B10**	7q33	0.43	4.73E-28	**7.28E-26**
**WNT4**	1p36.12	0.43	5.48E-28	**8.37E-26**
**SIDT1**	3q13.2	0.429	7.56E-28	**1.15E-25**
**PDE4D**	5q11.2-q12.1	0.428	8.97E-28	**1.35E-25**
**BARX2**	11q24.3	0.428	9.77E-28	**1.46E-25**
**GP9**	3q21.3	0.528	1.26E-27	**1.87E-25**
**RFX6**	6q22.1	0.527	1.69E-27	**2.48E-25**
**C4ORF19**	4p14	0.425	2.06E-27	**3.00E-25**
**LGALS4**	19q13.2	0.425	2.13E-27	**3.09E-25**
**GNE**	9p13.3	0.424	2.85E-27	**4.10E-25**
**PTGER4**	5p13.1	0.421	7.21E-27	**1.03E-24**
**LINC00930**	15q26.1	0.421	7.78E-27	**1.10E-24**
**F3**	1p21.3	0.421	8.71E-27	**1.23E-24**
**CHRM1**	11q12.3	0.421	9.01E-27	**1.26E-24**
**SH2D6**	2p11.2	0.419	1.30E-26	**1.80E-24**
**UGT1A10**	2q37.1	0.419	1.38E-26	**1.90E-24**
**BRINP3**	1q31.1	0.419	1.40E-26	**1.92E-24**
**INSM1**	20p11.23	0.418	2.23E-26	**3.03E-24**
**GRIN1**	9q34.3	0.416	3.45E-26	**4.66E-24**
**CCDC60**	12q24.23	0.416	3.68E-26	**4.94E-24**
**CLDN7**	17p13.1	0.415	5.02E-26	**6.70E-24**
**EPHA4**	2q36.1	0.415	5.49E-26	**7.28E-24**
**LIMA1**	12q13.12	0.414	5.58E-26	**7.35E-24**
**NR3C2**	4q31.23	0.414	6.20E-26	**8.11E-24**
**BTNL8**	5q35.3	0.414	6.25E-26	**8.13E-24**
**FAM174B**	15q26.1	0.414	7.20E-26	**9.30E-24**
**MUC5B**	11p15.5	0.413	7.83E-26	**1.01E-23**
**GPR15**	3q11.2	0.413	9.88E-26	**1.26E-23**
**CHST6**	16q23.1	0.412	1.00E-25	**1.27E-23**
**MPDU1**	17p13.1	0.412	1.25E-25	**1.58E-23**
**CDKN2B-AS1**	9p21.3	0.41	1.98E-25	**2.48E-23**
**TENT5C**	1p12	0.41	2.02E-25	**2.51E-23**
**RHBDL2**	1p34.3	0.409	3.02E-25	**3.74E-23**
**DYRK4**	12p13.32	0.409	3.20E-25	**3.93E-23**
**VWA3B**	2q11.2	0.406	7.39E-25	**8.96E-23**
**ACPP**	3q22.1	0.405	9.95E-25	**1.20E-22**
**SCNN1A**	12p13.31	0.404	1.16E-24	**1.38E-22**
**SPIB**	19q13.33	0.403	1.49E-24	**1.78E-22**

**CA7**	16q22.1	0.402	2.37E-24	**2.80E-22**
**DUSP4**	8p12	0.4	3.47E-24	**4.09E-22**
**TNFRSF11A**	18q21.33	0.4	3.86E-24	**4.52E-22**
**QSOX1**	1q25.2	0.399	4.40E-24	**5.12E-22**
**CD177**	19q13.31	0.398	6.58E-24	**7.61E-22**
**UGT2B15**	4q13.2	0.397	8.44E-24	**9.71E-22**
**CKAP4**	12q23.3	0.397	8.80E-24	**1.01E-21**
**GCNT3**	15q22.2	0.396	1.04E-23	**1.19E-21**
**COLCA1**	11q23.1	0.396	1.09E-23	**1.23E-21**
**HID1**	17q25.1	0.395	1.65E-23	**1.85E-21**
**MT1M**	16q13	0.394	1.90E-23	**2.12E-21**
**SLC35A1**	6q15	0.394	2.02E-23	**2.25E-21**
**MRAP2**	6q14.2	0.394	2.20E-23	**2.43E-21**
**CD55**	1q32.2	0.394	2.29E-23	**2.52E-21**
**HSD17B2**	16q23.3	0.393	2.35E-23	**2.57E-21**
**CDC42EP5**	19q13.42	0.393	2.99E-23	**3.25E-21**
**SCIN**	7p21.3	0.392	3.27E-23	**3.52E-21**
**ADGRF1**	6p12.3|6	0.391	4.05E-23	**4.34E-21**
**GPAT3**	4q21.23	0.391	4.70E-23	**5.00E-21**
**VNN1**	6q23.2	0.391	5.13E-23	**5.43E-21**
**UGT2B7**	4q13.2	0.39	5.40E-23	**5.69E-21**
**MFSD2A**	1p34.2	0.39	6.67E-23	**6.95E-21**
**STYK1**	12p13.2	0.389	7.72E-23	**8.01E-21**
**MCU**	10q22.1	0.388	9.60E-23	**9.86E-21**
**DMBT1**	10q26.13	0.388	1.06E-22	**1.08E-20**
**GPT**	8q24.3	0.388	1.12E-22	**1.14E-20**
**PIGR**	1q32.1	0.387	1.40E-22	**1.42E-20**
**SERPINB1**	6p25.2	0.387	1.46E-22	**1.47E-20**
**VSTM5**	11q21	0.386	1.63E-22	**1.63E-20**
**SLC35C1**	11p11.2	0.386	1.88E-22	**1.88E-20**
**PAPSS2**	10q23.2-q23.31	0.384	2.81E-22	**2.78E-20**
**RAB3B**	1p32.3	0.384	2.82E-22	**2.78E-20**
**FAM107B**	10p13	0.384	2.92E-22	**2.87E-20**

Table S2. The top 200 genes negatively correlated with *SPINK4*.

Correlated Gene	Cytoband	Spearman's Correlation	*p*-Value	*q*-Value
**DDX27**	20q13.13	-0.408	3.42E-25	**4.17E-23**
**CTNNBL1**	20q11.23	-0.392	3.17E-23	**3.43E-21**
**CSNK2A2**	16q21	-0.39	5.59E-23	**5.86E-21**
**STK4**	20q13.12	-0.389	8.30E-23	**8.57E-21**
**PHF20**	20q11.22-q11.23	-0.383	4.02E-22	**3.84E-20**
**PPP1R3D**	20q13.33	-0.377	1.90E-21	**1.69E-19**
**AMOTL2**	3q22.2	-0.376	2.30E-21	**2.03E-19**
**NORAD**	20q11.23	-0.375	3.49E-21	**3.03E-19**
**ANKRD27**	19q13.11	-0.374	3.94E-21	**3.39E-19**
**VAV2**	9q34.2	-0.373	5.03E-21	**4.25E-19**
**TOMM34**	20q13.12	-0.37	1.26E-20	**1.02E-18**
**TTPAL**	20q13.12	-0.369	1.64E-20	**1.30E-18**
**NFS1**	20q11.22	-0.369	1.64E-20	**1.30E-18**
**PFDN4**	20q13.2	-0.367	2.75E-20	**2.13E-18**
**TAF4**	20q13.33	-0.366	3.16E-20	**2.44E-18**

**NCOA6**	20q11.22	-0.366	3.36E-20	**2.58E-18**
**SLC5A6**	2p23.3	-0.364	5.79E-20	**4.29E-18**
**ZNF251**	8q24.3	-0.363	7.49E-20	**5.49E-18**
**CEP250**	20q11.22	-0.363	7.73E-20	**5.65E-18**
**ASXL1**	20q11.21	-0.361	1.03E-19	**7.42E-18**
**ADNP**	20q13.13	-0.36	1.62E-19	**1.14E-17**
**LSM14B**	20q13.33	-0.359	1.89E-19	**1.32E-17**
**RBM39**	20q11.22	-0.359	2.12E-19	**1.47E-17**
**TCFL5**	20q13.33	-0.356	3.54E-19	**2.42E-17**
**C11ORF95**	11q13.1	-0.356	3.66E-19	**2.47E-17**
**YTHDF1**	20q13.33	-0.354	6.09E-19	**4.00E-17**
**ACTR5**	20q11.23	-0.354	7.15E-19	**4.68E-17**
**PIPOX**	17q11.2	-0.353	7.98E-19	**5.20E-17**
**RPIA**	2p11.2	-0.353	8.65E-19	**5.60E-17**
**NDRG3**	20q11.23	-0.352	1.01E-18	**6.50E-17**
**DKC1**	Xq28	-0.352	1.10E-18	**7.07E-17**
**STAU1**	20q13.13	-0.351	1.41E-18	**8.75E-17**
**NCBP2**	3q29	-0.35	1.74E-18	**1.07E-16**
**PRPF6**	20q13.33	-0.347	3.30E-18	**1.98E-16**
**POFUT1**	20q11.21	-0.346	3.98E-18	**2.38E-16**
**ZPR1**	11q23.3	-0.346	4.08E-18	**2.43E-16**
**NELFCD**	20q13.32	-0.346	4.37E-18	**2.59E-16**
**STX16**	20q13.32	-0.346	4.75E-18	**2.80E-16**
**YAP1**	11q22.1	-0.343	7.81E-18	**4.50E-16**
**LRP11**	6q25.1	-0.341	1.29E-17	**7.27E-16**
**RTF2**	20q13.31	-0.34	1.84E-17	**1.03E-15**
**EIF2S2**	20q11.22	-0.339	2.05E-17	**1.15E-15**
**CIAO1**	2q11.2	-0.339	2.30E-17	**1.28E-15**
**TM9SF4**	20q11.21	-0.338	2.68E-17	**1.49E-15**
**RALGAPB**	20q11.23	-0.338	2.80E-17	**1.55E-15**
**TTI1**	20q11.23	-0.337	3.35E-17	**1.83E-15**
**HSPH1**	13q12.3	-0.336	4.51E-17	**2.41E-15**
**NCL**	2q37.1	-0.335	5.44E-17	**2.87E-15**
**ELMO2**	20q13.12	-0.335	5.76E-17	**3.02E-15**
**SLC4A1AP**	2p23.3	-0.333	7.99E-17	**4.13E-15**
**NCK2**	2q12.2	-0.333	8.80E-17	**4.54E-15**
**GID8**	20q13.33	-0.332	1.02E-16	**5.24E-15**
**REEP1**	2p11.2	-0.332	1.18E-16	**6.02E-15**
**CDK5RAP1**	20q11.21	-0.33	1.73E-16	**8.70E-15**
**LDLRAD3**	11p13	-0.33	1.77E-16	**8.85E-15**
**AATF**	17q12	-0.328	2.34E-16	**1.16E-14**
**LOC646762**	7p14.3	-0.328	2.60E-16	**1.27E-14**
**MAPRE1**	20q11.21	-0.328	2.84E-16	**1.39E-14**
**LY6G6D**	6p21.33	-0.326	4.44E-16	**2.14E-14**
**HABP4**	9q22.32	-0.325	5.07E-16	**2.43E-14**
**SCRN1**	7p14.3	-0.325	5.11E-16	**2.44E-14**
**NCOA5**	20q13.12	-0.325	5.19E-16	**2.47E-14**
**DPM1**	20q13.13	-0.324	5.55E-16	**2.63E-14**
**ZNF23**	16q22.2	-0.324	6.61E-16	**3.11E-14**
**CHMP4B**	20q11.22	-0.323	7.03E-16	**3.29E-14**
**DIDO1**	20q13.33	-0.323	7.66E-16	**3.57E-14**

**CBX1**	17q21.32	-0.322	9.92E-16	**4.58E-14**
**DYNLRB1**	20q11.22	-0.322	1.02E-15	**4.71E-14**
**SMYD5**	2p13.2	-0.32	1.48E-15	**6.76E-14**
**RAB22A**	20q13.32	-0.32	1.50E-15	**6.82E-14**
**RBM26**	13q31.1	-0.319	1.62E-15	**7.36E-14**
**NOP58**	2q33.1	-0.319	1.75E-15	**7.90E-14**
**ZNF696**	8q24.3	-0.319	1.81E-15	**8.15E-14**
**ZGPAT**	20q13.33	-0.319	1.98E-15	**8.91E-14**
**HSP90AB1**	6p21.1	-0.318	2.34E-15	**1.05E-13**
**RNF219**	13q31.1	-0.318	2.47E-15	**1.10E-13**
**RAE1**	20q13.31	-0.317	2.75E-15	**1.21E-13**
**YEATS2**	3q27.1	-0.317	2.98E-15	**1.30E-13**
**NODAL**	10q22.1	-0.316	3.08E-15	**1.34E-13**
**WBP4**	13q14.11	-0.316	3.47E-15	**1.50E-13**
**MPHOSPH10**	2p13.3	-0.316	3.49E-15	**1.51E-13**
**SLC6A4**	17q11.2	-0.313	5.79E-15	**2.45E-13**
**LRRC37A3**	17q24.1	-0.313	5.91E-15	**2.50E-13**
**JADE3**	Xp11.3	-0.313	5.96E-15	**2.51E-13**
**DDX10**	11q22.3	-0.313	6.01E-15	**2.53E-13**
**BCL2L1**	20q11.21	-0.313	6.34E-15	**2.66E-13**
**NOL10**	2p25.1	-0.313	6.85E-15	**2.87E-13**
**RNF6**	13q12.13	-0.313	7.04E-15	**2.94E-13**
**SS18L1**	20q13.33	-0.312	7.59E-15	**3.15E-13**
**PIGU**	20q11.22	-0.311	8.76E-15	**3.62E-13**
**HSPD1**	2q33.1	-0.311	8.87E-15	**3.66E-13**
**FXR1**	3q26.33	-0.31	1.11E-14	**4.54E-13**
**WDR35**	2p24.1	-0.31	1.16E-14	**4.70E-13**
**CCT6A**	7p11.2	-0.31	1.27E-14	**5.14E-13**
**NSRP1**	17q11.2	-0.31	1.30E-14	**5.26E-13**
**PLAGL2**	20q11.21	-0.309	1.33E-14	**5.36E-13**
**NFE2L3P2**	17q21.32	-0.309	1.34E-14	**5.39E-13**
**DHX35**	20q11.23-q12	-0.309	1.36E-14	**5.46E-13**
**MAB21L4**	2q37.3	-0.309	1.42E-14	**5.69E-13**
**XPO5**	6p21.1	-0.309	1.43E-14	**5.70E-13**
**FAM217B**	20q13.33	-0.308	1.65E-14	**6.54E-13**
**USP27X**	Xp11.23	-0.307	1.98E-14	**7.81E-13**
**SPAG9**	17q21.33	-0.307	1.98E-14	**7.81E-13**
**CLASP1**	2q14.2-q14.3	-0.307	2.03E-14	**7.99E-13**
**PHACTR3**	20q13.32-q13.33	-0.306	2.50E-14	**9.76E-13**
**ATIC**	2q35	-0.306	2.76E-14	**1.07E-12**
**SMG7**	1q25.3	-0.306	2.84E-14	**1.10E-12**
**PROSER1**	13q13.3	-0.306	2.87E-14	**1.11E-12**
**MPP1**	Xq28	-0.305	3.00E-14	**1.16E-12**
**FMR1**	Xq27.3	-0.305	3.22E-14	**1.24E-12**
**TRMT11**	6q22.32	-0.305	3.42E-14	**1.31E-12**
**TOP1**	20q12	-0.305	3.59E-14	**1.37E-12**
**PABPC1L**	20q13.12	-0.305	3.60E-14	**1.37E-12**
**ZSWIM3**	20q13.12	-0.304	4.32E-14	**1.63E-12**
**WNT11**	11q13.5	-0.303	4.45E-14	**1.67E-12**
**PPP1R9B**	17q21.33	-0.303	4.67E-14	**1.74E-12**
**SORBS1**	10q24.1	-0.303	5.14E-14	**1.90E-12**

**RGS9BP**	19q13.11	-0.302	5.54E-14	**2.03E-12**
**CUL4A**	13q34	-0.302	5.71E-14	**2.09E-12**
**CENPJ**	13q12.12-q12.13	-0.302	5.98E-14	**2.18E-12**
**PALM2-AKAP2**	9q31.3	-0.302	6.08E-14	**2.22E-12**
**ZNF608**	5q23.2	-0.302	6.39E-14	**2.32E-12**
**LY6G6E**	6p21.33	-0.301	6.61E-14	**2.40E-12**
**LTV1**	6q24.2	-0.301	6.76E-14	**2.44E-12**
**POLR1A**	2p11.2	-0.301	7.00E-14	**2.52E-12**
**WDR53**	3q29	-0.301	7.08E-14	**2.54E-12**
**SHROOM4**	Xp11.22	-0.301	7.61E-14	**2.71E-12**
**MORC2**	22q12.2	-0.301	7.75E-14	**2.75E-12**
**SRCIN1**	17q12	-0.301	8.03E-14	**2.84E-12**
**RPRD1B**	20q11.23	-0.3	8.77E-14	**3.09E-12**
**ETNK2**	1q32.1	-0.3	8.79E-14	**3.09E-12**
**TOP1MT**	8q24.3	-0.3	9.41E-14	**3.30E-12**
**CSE1L**	20q13.13	-0.3	9.82E-14	**3.42E-12**
**BCL11A**	2p16.1	-0.299	1.12E-13	**3.86E-12**
**BRD7**	16q12.1	-0.299	1.13E-13	**3.92E-12**
**R3HDML**	20q13.12	-0.299	1.17E-13	**4.01E-12**
**C11ORF74**	11p12	-0.298	1.22E-13	**4.16E-12**
**NFE2L3**	7p15.2	-0.298	1.26E-13	**4.27E-12**
**SMC3**	10q25.2	-0.298	1.34E-13	**4.52E-12**
**CEL**	9q34.13	-0.298	1.40E-13	**4.70E-12**
**CTSV**	9q22.33	-0.297	1.50E-13	**5.01E-12**
**UPF3A**	13q34	-0.297	1.66E-13	**5.50E-12**
**TGIF2**	20q11.23	-0.297	1.69E-13	**5.59E-12**
**CHD6**	20q12	-0.296	1.79E-13	**5.93E-12**
**WWC3**	Xp22.2	-0.296	1.82E-13	**6.02E-12**
**SLC2A12**	6q23.2	-0.296	1.85E-13	**6.10E-12**
**GTF2IRD1**	7q11.23	-0.296	1.87E-13	**6.15E-12**
**ABI2**	2q33.2	-0.296	1.96E-13	**6.42E-12**
**SUPT7L**	2p23.3	-0.296	2.05E-13	**6.70E-12**
**TULP4**	6q25.3	-0.296	2.09E-13	**6.83E-12**
**ISY1**	3q21.3	-0.296	2.11E-13	**6.88E-12**
**BIN1**	2q14.3	-0.295	2.19E-13	**7.12E-12**
**STIP1**	11q13.1	-0.295	2.25E-13	**7.32E-12**
**OSER1**	20q13.12	-0.295	2.40E-13	**7.78E-12**
**AAR2**	20q11.23	-0.295	2.58E-13	**8.32E-12**
**UXS1**	2q12.2	-0.294	2.62E-13	**8.42E-12**
**BCORL1**	Xq26.1	-0.294	2.73E-13	**8.74E-12**
**SNX25**	4q35.1	-0.294	2.86E-13	**9.15E-12**
**ST6GAL1**	3q27.3	-0.294	2.90E-13	**9.26E-12**
**RTKN**	2p13.1	-0.294	2.97E-13	**9.43E-12**
**CTSZ**	20q13.32	-0.294	3.02E-13	**9.56E-12**
**UBE2V1**	20q13.13	-0.294	3.04E-13	**9.60E-12**
**PLCG1**	20q12	-0.294	3.11E-13	**9.78E-12**
**CEP63**	3q22.2	-0.293	3.25E-13	**1.02E-11**
**GDPD5**	11q13.4-q13.5	-0.293	3.63E-13	**1.12E-11**
**UPF3B**	Xq24	-0.293	3.83E-13	**1.18E-11**
**PTPN1**	20q13.13	-0.292	4.30E-13	**1.32E-11**
**GTF2F2**	13q14.12-q14.13	-0.292	4.42E-13	**1.35E-11**

**RMND5A**	2p11.2	-0.292	4.43E-13	**1.35E-11**
**OSBPL2**	20q13.33	-0.292	4.54E-13	**1.38E-11**
**PALD1**	10q22.1	-0.292	4.61E-13	**1.40E-11**
**PDCL3**	2q11.2	-0.292	4.63E-13	**1.40E-11**
**RNF43**	17q22	-0.292	4.64E-13	**1.40E-11**
**DNAJC7**	17q21.2	-0.291	4.65E-13	**1.40E-11**
**FARP1**	13q32.2	-0.291	4.77E-13	**1.43E-11**
**ZNF250**	8q24.3	-0.291	4.91E-13	**1.47E-11**
**NUFIP1**	13q14.12	-0.291	5.07E-13	**1.52E-11**
**SSB**	2q31.1	-0.291	5.10E-13	**1.52E-11**
**ATR**	3q23	-0.291	5.19E-13	**1.55E-11**
**NOL8**	9q22.31	-0.291	5.30E-13	**1.58E-11**
**CPNE1**	20q11.22	-0.291	5.50E-13	**1.63E-11**
**ZBTB2**	6q25.1	-0.291	5.56E-13	**1.65E-11**
**HNRNPH3**	10q21.3	-0.291	5.60E-13	**1.66E-11**
**SNRNP200**	2q11.2	-0.29	5.69E-13	**1.68E-11**
**TPX2**	20q11.21	-0.29	6.17E-13	**1.81E-11**
**MRGBP**	20q13.33	-0.29	6.20E-13	**1.81E-11**
**NRXN2**	11q13.1	-0.29	6.53E-13	**1.91E-11**
**E2F6**	2p25.1	-0.289	7.29E-13	**2.11E-11**
**DPF2**	11q13.1	-0.288	8.26E-13	**2.38E-11**
**UTP14A**	Xq26.1	-0.288	8.83E-13	**2.53E-11**
**DNMT3B**	20q11.21	-0.288	8.85E-13	**2.54E-11**
**PRPF3**	1q21.2	-0.288	9.48E-13	**2.70E-11**
**USP36**	17q25.3	-0.288	9.60E-13	**2.74E-11**
**QTRT2**	3q13.31	-0.288	9.68E-13	**2.75E-11**
**ZMYND8**	20q13.12	-0.287	9.96E-13	**2.83E-11**
**NCOA3**	20q13.12	-0.287	1.00E-12	**2.83E-11**
**ANXA9**	1q21.3	-0.287	1.01E-12	**2.84E-11**
**ANKRD13B**	17q11.2	-0.287	1.01E-12	**2.85E-11**
**ZC3H15**	2q32.1	-0.287	1.03E-12	**2.89E-11**
**PIP4K2B**	17q12	-0.287	1.07E-12	**3.00E-11**
